# Head-to-head evaluation of [^18^F]FDG and [^68^ Ga]Ga-DOTA-FAPI-04 PET/CT in recurrent soft tissue sarcoma

**DOI:** 10.1007/s00259-022-05700-4

**Published:** 2022-02-03

**Authors:** Bingxin Gu, Xin Liu, Shuoer Wang, Xiaoping Xu, Xiaosheng Liu, Silong Hu, Wangjun Yan, Zhiguo Luo, Shaoli Song

**Affiliations:** 1 Department of Nuclear Medicine, Fudan University Shanghai Cancer Center, Shanghai, China; 2 Department of Oncology, Shanghai Medical College, Fudan University, Shanghai, China; 3 Center for Biomedical Imaging, Fudan University, Shanghai, China; 4 Shanghai Engineering Research Center of Molecular Imaging Probes, Shanghai, China; 5 Key Laboratory of Nuclear Physics and Ion-Beam Application (MOE), Fudan University, Shanghai, China; 6 Department of Medical Oncology, Fudan University Shanghai Cancer Center, Shanghai, China; 7 Department of Musculoskeletal Tumor, Fudan University Shanghai Cancer Center, Shanghai, China

**Keywords:** FAPI, FDG, Soft tissue sarcoma, PET/CT, SUV

## Abstract

**Purpose:**

We aimed to evaluate the value of [^68^ Ga]Ga-DOTA-FAPI-04 PET/CT for the diagnosis of recurrent soft tissue sarcoma (STS), compared with [^18^F]FDG PET/CT.

**Methods:**

A total of 45 patients (21 females and 24 males; median age, 46 years; range, 18–71 years) with 13 subtypes of STS underwent [^18^F]FDG and [^68^ Ga]Ga-DOTA-FAPI-04 PET/CT examination within 1 week for assessment local relapse or distant metastasis. Positive lesions on PET/CT images were verified by biopsy or 3-month follow-up. Wilcoxon matched-pairs signed-rank test was used to compare the semiquantitative values (SUV_max_ and TBR) of [^18^F]FDG and [^68^ Ga]Ga-DOTA-FAPI-04 in tumor lesions, and McNemar test was applied to test for differences of both tracers.

**Results:**

Among the 45 patients, 282 local relapses and distant metastases were identified. Compared to [^18^F]FDG, [^68^ Ga]Ga-DOTA-FAPI-04 PET/CT detected more lesions (275 vs. 186) and outperformed in sensitivity, specificity, PPV, NPV, and accuracy for the diagnosis of recurrent lesions (*P* < 0.001). [^68^ Ga]Ga-DOTA-FAPI-04 demonstrated significantly higher values of SUV_max_ and TBR than [^18^F]FDG PET/CT in liposarcoma (*P* = 0.011 and *P* < 0.001, respectively), malignant solitary fibrous tumor (MSFT) (*P* < 0.001 and *P* < 0.001, respectively), and interdigitating dendritic cell sarcoma (IDCS) (*P* < 0.001and *P* < 0.001, respectively). While mean SUV_max_ and TBR presented favorable uptake of [^18^F]FDG over [^68^ Ga]Ga-DOTA-FAPI-04 in undifferentiated pleomorphic sarcoma (UPS) (*P* = 0.003 and *P* < 0.001, respectively) and rhabdomyosarcoma (RMS) (*P* < 0.001 and *P* < 0.001, respectively).

**Conclusion:**

[^68^ Ga]Ga-DOTA-FAPI-04 PET/CT is a promising new imaging modality for recurrent surveillance of STS, and compares favorably with [^18^F]FDG for identifying recurrent lesions of liposarcoma, MSFT, and IDCS.

**Supplementary Information:**

The online version contains supplementary material available at 10.1007/s00259-022-05700-4.

## Introduction

Soft tissue sarcomas (STS) are rare and heterogeneous tumors, which contain more than 50 different histologic subtypes according to the World Health Organization (WHO) classification [1]. The prognosis of metastatic STS is dismal, with a median overall survival (OS) of 8–12 months [2]. Thus, optimal imaging of STS is crucial for accurately restaging and detecting local relapse and/or distant metastasis as early and as completely as possible. The most frequent metastatic sites of STS are the lung, followed by bone and lymph nodes [3]. Computed tomography (CT) and magnetic resonance imaging (MRI) serve as the routine means for local relapsed surveillance. But for detecting distant metastasis, [^18^F]-fluorodeoxyglucose ([^18^F]FDG) positron emission tomography/computed tomography (PET/CT) shows higher sensitivity and accuracy [4]. Furthermore, [^18^F]FDG PET/CT is useful for initial staging and restaging, evaluation of treatment response, and predicting treatment efficacy and clinical outcome for STS [5]. However, due to lack of sensitivity among some subtypes of sarcomas, particularly low-grade sarcomas, [^18^F]FDG PET/CT is not generally recommended for the management of sarcomas [6–8].

Recently, new development of PET tracers targeting fibroblast activation protein (FAP), [^68^ Ga]-fibroblast activation protein inhibitor (FAPI), had shown promising results in imaging of sarcomas [9]. FAP is a type II membrane-bound glycoprotein belonging to the dipeptidyl peptidase 4 family, which has both dipeptidyl peptidase and endopeptidase activity. FAP plays a pivotal role in tumor microenvironment, including reduced levels of anti-angiogenic factors, elevated levels of transforming growth factor β, and affected matrix processing enzymes [10]. FAP is overexpressed in cancer-associated fibroblasts (CAFs) in the stroma of more than 90% of epithelial carcinomas [11] and many subtypes of STS (e.g., fibrosarcoma, malignant fibrous histiocytoma, and liposarcoma) [12, 13]. In addition to diagnostic imaging, FAP is also considered as a promising target for delivering therapeutic nuclide [14]. This may provide a new approach for recurrent STS to improve survival. Thus, the expression of FAP on different STS needs to be identified.

Inspired by the promising results of [^68^ Ga]Ga-DOTA-FAPI-04 imaging on many epithelial carcinomas [9, 15, 16], we hypothesized that [^68^ Ga]Ga-DOTA-FAPI-04 would outperform [^18^F]FDG in recurrent surveillance of STS. Herein, in this study, we aimed to investigate the potential usefulness of [^68^ Ga]Ga-DOTA-FAPI-04 PET/CT for the diagnosis of recurrent lesions in patients with STS, compared with [^18^F]FDG PET/CT. The primary objective of this study was the comparison of uptake of [^18^F]FDG and [^68^ Ga]Ga-DOTA-FAPI-04 by different histological STS subtypes. Secondary objectives were the comparison by grading and by lesion location.

## Methods

### Patient selection

This prospective clinical trial (CFFSTS Trial, ChiCTR2100053984, Chinese Clinical Trial Registry) was conducted in Fudan University Shanghai Cancer Center to compare the diagnostic ability of [^68^ Ga]Ga-DOTA-FAPI-04 and [^18^F]FDG PET/CT in patients with STS from May 2020. To further investigate the role of [^68^ Ga]Ga-DOTA-FAPI-04 in recurrent STS, inclusion criteria were as follows: (i) pathologically confirmed STS; (ii) patients were suspected recurrence after radical treatment. The exclusion criteria were (i) patients without recurrence; (ii) patients with two or more malignant tumor history; and (iii) patients unwilling to take [^18^F]FDG and [^68^ Ga]Ga-DOTA-FAPI-04 PET/CT. Data including demographics, tumor characteristics, and treatment information were collected from the medical records. This prospective study was approved by Fudan University Shanghai Cancer Center Institutional Review Board (ID 2,004,216–25) and conducted in accordance with the 1964 Declaration of Helsinki and its later amendments or comparable ethical standards. Informed consents to undergo [^18^F]FDG and [^68^ Ga]Ga-DOTA-FAPI-04 PET/CT were obtained from all enrolled patients.

### Radiopharmaceuticals and PET/CT scanning procedure

[^18^F]FDG was produced automatically using Explora FDG_4_ module with cyclotron (Siemens CTI RDS Eclips ST, Knoxville, Tennessee, USA) in our center. DOTA-FAPI-04 was obtained commercially (Jiangsu Huayi Technology CO., LTD, Jiangsu, China). DOTA-FAPI-04 was radiolabeled with ^68^ Ga according to Lindner et al. [14]. Briefly, the DOTA-FAPI-04 and ^68^ Ga-solution (elution with 0.5 M HCl) were mixed with sodium acetate, and the pH was maintained about 4.5. Then the reaction mixture was heated to 100 °C for 20 min. The [^68^ Ga]Ga-DOTA-FAPI-04 was obtained by solid phase extraction. Radiochemical purity of [^18^F]FDG and [^68^ Ga]Ga-DOTA-FAPI-04 was both over 95%.

[^18^F]FDG and [^68^ Ga]Ga-DOTA-FAPI-04 PET/CT scans were performed within 1 week. For [^18^F]FDG PET/CT scanning, patients fasted at least 6 h, maintaining venous blood glucose levels under 10 mmol/L prior to [^18^F]FDG administration. But this was not necessary for [^68^ Ga]Ga-DOTA-FAPI-04 PET/CT scanning. After injecting with 242.62 ± 43.83 MBq of [^18^F]FDG or 147.69 ± 21.55 MBq of [^68^ Ga]Ga-DOTA-FAPI-04, patients were kept in a quiet environment for approximately 60 min prior to examination. No adverse or clinically detectable pharmacological effects were observed in any of these patients. All images were obtained on a Biograph mCT Flow scanner (Siemens Medical Solutions). Low-dose CT scanning was performed firstly for location: scanning ranging from the proximal thighs or feet to head, with 120 kV, 100 mAs, CARE Dose4D, slice thickness 3 mm, increment 2 mm, pitch 1.0, rotation time 0.5 s, and soft-tissue reconstruction kernel. Immediately after CT scanning, a PET emission scan that covered the corresponding field of CT was acquired in 3-dimensional mode using FlowMotion with a speed of 2. The emission data were corrected for random scatter and decay. PET image datasets were reconstructed iteratively using an ordered-subset expectation maximization iterative reconstruction by applying CT data for attenuation correction. Fusion images were reviewed and manipulated on a multimodality computer platform (Syngo, Siemens, Knoxville, Tennessee, USA). Two experienced nuclear medicine physicians analyzed and interpreted the images independently, and they reached a consensus in case of inconsistency.

Increased radioactivity of relapsed or metastatic lesions compared with the uptake of surrounding normal tissue was defined as being positive, verified by biopsy or 3-month follow-up. Lesions were considered malignant during follow-up based on (i) typical malignant features (i.e., mass, abnormal density, poor circumscription, and destruction), and (ii) a significant reduction or progression in size after anticancer treatment confirmed by follow-up imaging (i.e., CT and MRI) according to RECIST 1.1 [17]. For quantitative analysis, maximum and mean of standardized uptake value (SUV) normalized to body weight were manually computed for tumor lesions and healthy tissues by drawing a 3-dimensional volume of interest, respectively. Meanwhile, tumor-to-background ratio (TBR) for tumor lesions was calculated according to the formula: TBR = tSUVmax/bSUVmean, where tSUVmax is the maximum SUV of tumor lesion, and bSUVmean is the mean SUV of normal tissue.

### Statistical analyses

All statistical analyses were performed using SPSS 25.0 (IBM, Armonk, NY, USA). Mean with standard deviation or median with range was used to describe continuous characteristics. Sensitivity, specificity, positive predictive value (PPV), negative predictive value (NPV), and accuracy of [^18^F]FDG and [^68^ Ga]Ga-DOTA-FAPI-04 were determined, and McNemar test was applied to test for differences of both tracers. To compare the semiquantitative values (SUV_max_ and TBR) of [^18^F]FDG and [^68^ Ga]Ga-DOTA-FAPI-04 in tumor lesions, Wilcoxon matched-pairs signed-rank test was used. Two-tailed *p* < 0.05 was considered statistically significant.

## Results

### Patients

**Fig. 1:**
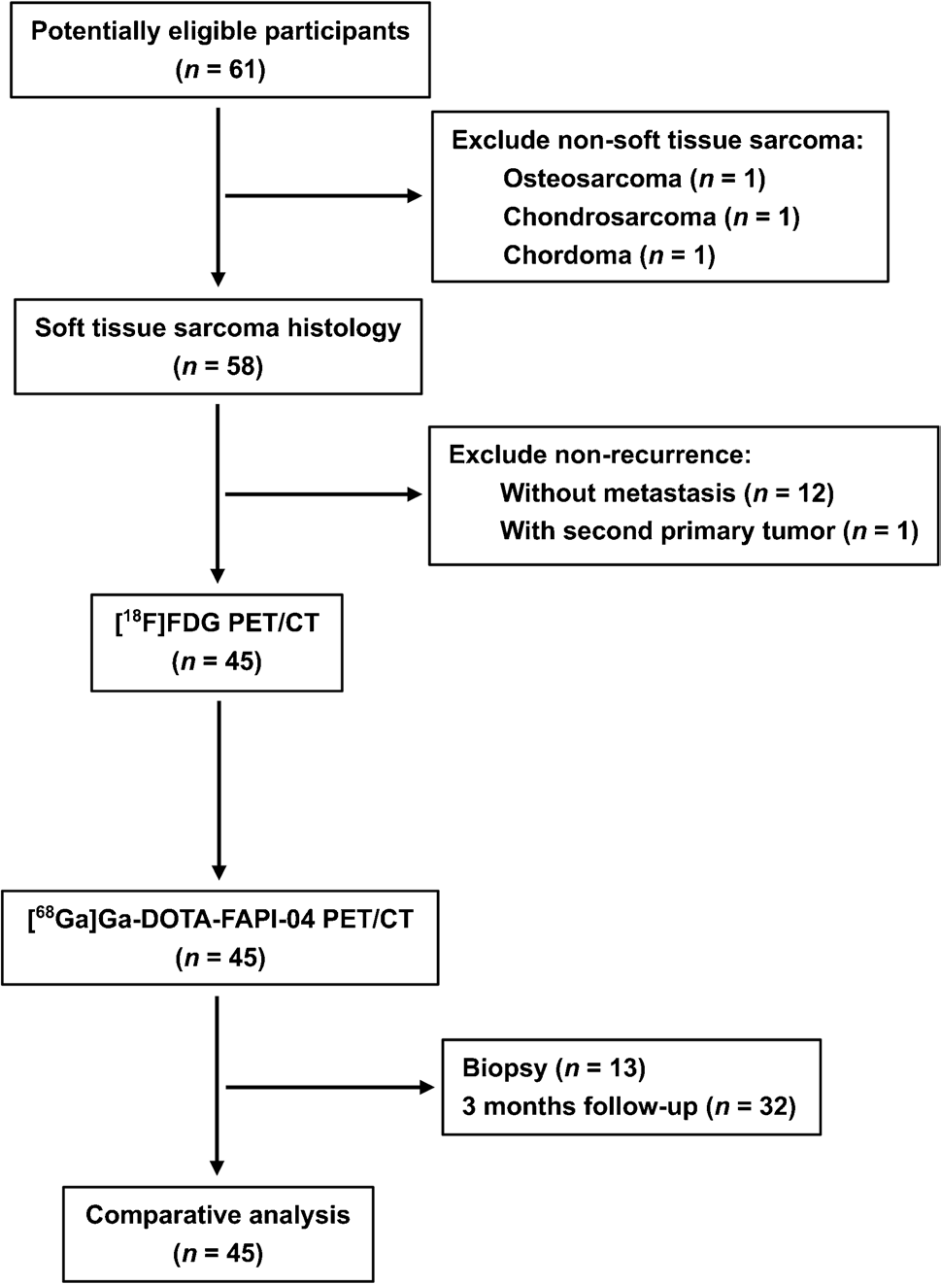
Flowchart of patient selection

From May 2020 to May 2021, 45 patients (21 females and 24 males; median age = 46 years, range, 18–71 years) were consecutively enrolled in this study (Fig. 1). All patients were diagnosed with STS and got radical treatment (e.g., surgery, radiotherapy, chemotherapy, or combination therapy) before PET/CT scans. Diagnostic CT or MRI was performed in 30 out of 45 patients prior to PET/CT scans, and positive findings were observed in 29 patients (Table 1).

**Table 1:** Detailed clinical characteristics of the patients

Patient	Gender	Age	Histology	Initial treatment		Diagnostic CT/MRI before PET/CT scan*	Restaging	
				Localization	Grading		FDG	FAPI-04
1	F	51	UPS	Thigh	G3	CT +	LR + DM	LR + DM
2	M	58	UPS	Retroperitoneum	G3	/	LR + DM	LR + DM
3	M	71	UPS	Retroperitoneum	G3	CT +	LR + DM	LR + DM
4	M	53	UPS	Neck	G3	MRI +	LR	LR
5	F	46	UPS	Small intestine	G3	/	LR	LR
6	M	63	UPS	Retroperitoneum	G3	/	LR + DM	LR + DM
7	M	22	UPS	Inguinal	G3	/	LR	LR
8	F	64	Well-differentiated liposarcoma	Retroperitoneum	G1	CT +	LR + DM	LR + DM
9	F	59	Pleomorphic liposarcoma	Lower leg	G3	CT +	LR + DM	LR + DM
10	M	67	Well-differentiated liposarcoma	Retroperitoneum	G1	/	LR	LR
11	M	70	Dedifferentiated liposarcoma	Thigh	G3	/	DM	DM
12	F	39	Dedifferentiated liposarcoma	Retroperitoneum	G3	CT +	LR	LR
13	F	36	Well-differentiated liposarcoma	Retroperitoneum	G1	MRI +	Non-recurrence	LR
14	M	34	Synovial sarcoma	Postmediastinum	G3	CT +	LR	LR
15	F	55	Synovial sarcoma	Kidney	G3	/	LR + DM	LR + DM
16	M	23	Synovial sarcoma	Lung	G3	CT +	LR	LR
17	M	38	Synovial sarcoma	Abdomen	G3	MRI +	LR	LR
18	F	65	Synovial sarcoma	Lower leg	G3	CT +	LR	LR
19	M	41	Synovial sarcoma	Kidney	G3	MRI +	LR	LR + DM
20	M	20	RMS	Cheek	G3	CT +	DM	DM
21	F	29	RMS	Perineum	G3	MRI +	LR + DM	LR + DM
22	F	19	RMS	Nasal cavity	G3	CT -	DM	DM
23	M	25	RMS	Nasal cavity	G3	MRI +	DM	DM
24	F	18	RMS	Perianal region	G3	MRI +	LR + DM	LR + DM
25	M	47	MSFT	Thigh	G2	CT +	LR + DM	LR + DM
26	M	69	MSFT	Retroperitoneum	Unknown	/	LR	LR + DM
27	M	49	MSFT	Neck	G1	/	DM	DM
28	F	65	MSFT	Uterus	G2	CT +	LR + DM	LR + DM
29	M	19	Ewing sarcoma	Thigh	G3	MRI +	LR	LR
30	M	20	Ewing sarcoma	Buttock	G3	/	DM	DM
31	M	25	Ewing sarcoma	Pelvic cavity	G3	/	LR	LR
32	F	29	Ewing sarcoma	Perianal region	G3	MRI +	LR	LR
33	F	60	Leiomyosarcoma	Uterus	Unknown	CT +	DM	DM
34	F	55	Leiomyosarcoma	Uterus	Unknown	CT +	DM	DM
35	F	47	Leiomyosarcoma	Retroperitoneum	G2	/	LR + DM	LR + DM
36	F	51	Leiomyosarcoma	Uterus	G2	MRI +	DM	DM
37	M	28	Myxofibrosarcoma	Shoulder	G3	CT +	DM	DM
38	M	46	Myxofibrosarcoma	Waist	G2	/	DM	DM
39	M	33	Myxofibrosarcoma	Buttock	G1	/	LR	LR
40	F	47	ASPS	Small intestine	G3	MRI +	LR + DM	LR + DM
41	F	19	ASPS	Abdomen	G3	MRI +	LR + DM	LR + DM
42	F	66	Epithelioid sarcoma	Inguinal	G3	/	LR	LR
43	M	23	Aggressive fibromatosis	Pelvic cavity	Unknown	CT +	LR	LR
44	F	44	FDCS	Liver	Unknown	CT +	LR + DM	LR + DM
45	M	48	IDCS	Liver	Unknown	CT +	Non-recurrence	LR + DM

^*^ “ + ” means diagnostic CT/MRI could detect the relapsed or metastatic lesions, and “-” indicates diagnostic CT/MRI could not detect the relapsed or metastatic lesions. *F*, female; *M*, male; *UPS*, undifferentiated pleomorphic sarcoma; *RMS*, rhabdomyosarcoma; *MSFT*, malignant solitary fibrous tumor; *ASPS*, alveolar soft part sarcoma; *FDCS*, follicular dendritic cell sarcoma; *IDCS*, interdigitating dendritic cell sarcoma; *LR*, local relapse; *DM*, distant metastasis

### Comparison of [^18^F]FDG and [^68^ Ga]Ga-DOTA-FAPI-04 PET/CT based on different subtypes of recurrent STS

**Fig. 2:**
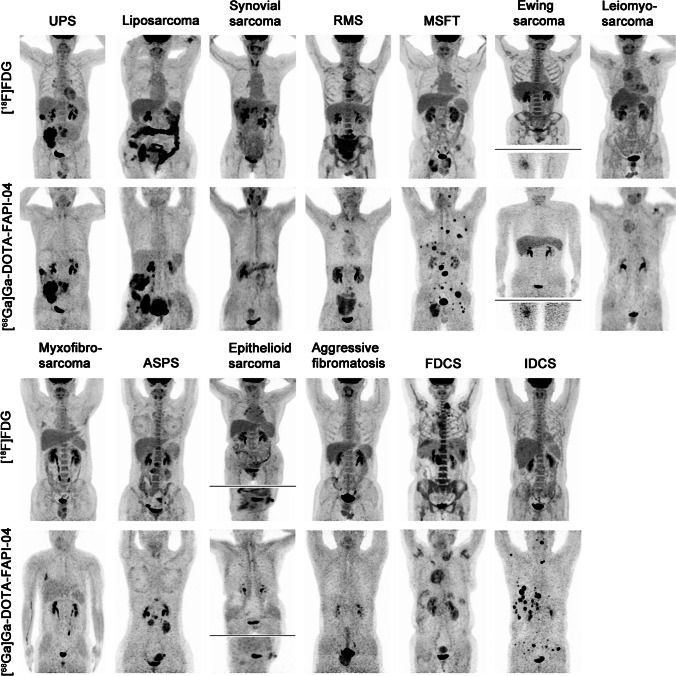
MIP images of [^18^F]FDG PET/CT and [^68^ Ga]Ga-DOTA-FAPI-04 PET/CT in patients reflecting 13 different representative recurrent STS entities. MIP, maximum-intensity projection; STS, soft tissue sarcoma; UPS, undifferentiated pleomorphic sarcoma; RMS, rhabdomyosarcoma; MSFT, malignant solitary fibrous tumor; ASPS, alveolar soft part sarcoma; FDCS, follicular dendritic cell sarcoma; IDCS, interdigitating dendritic cell sarcoma

**Fig. 3:**
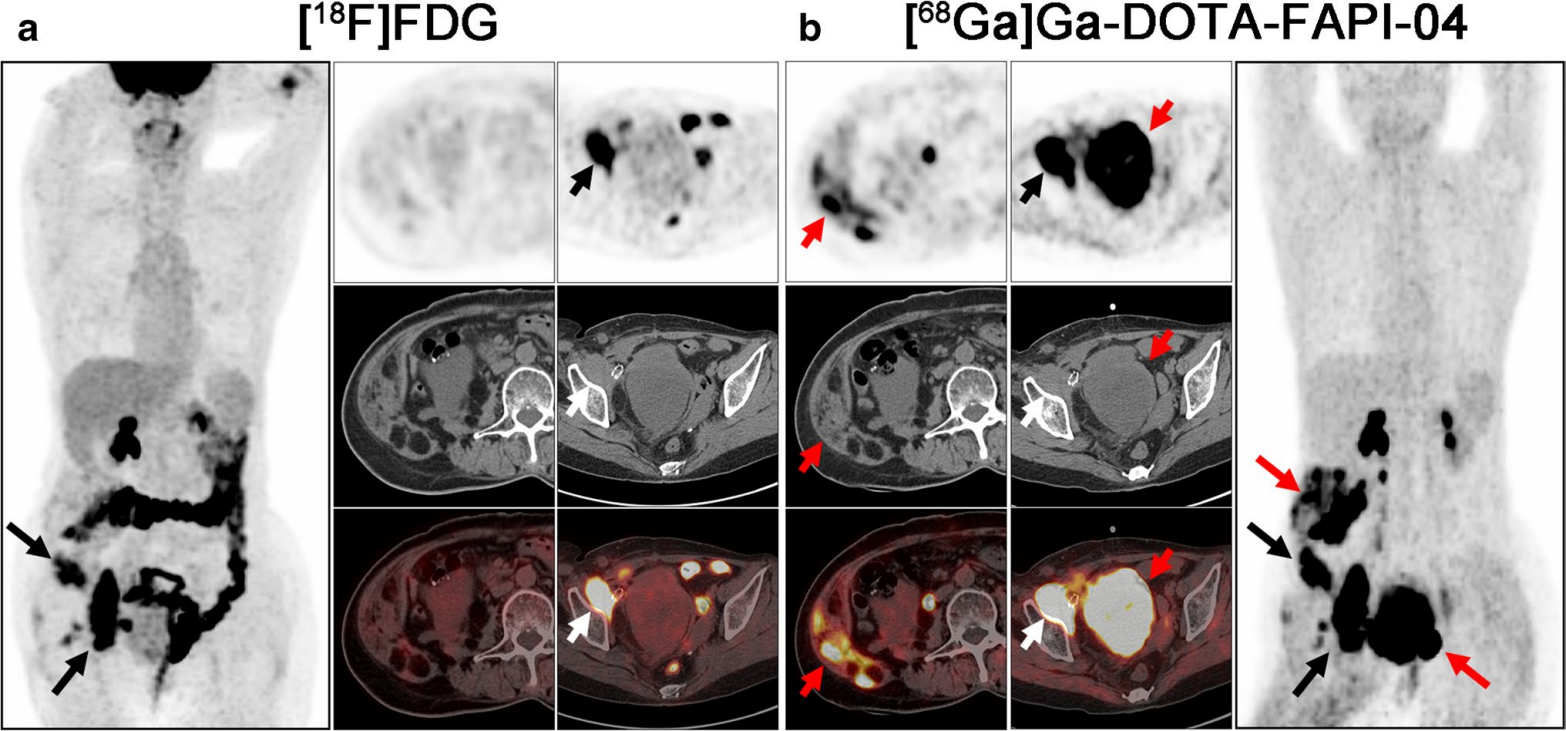
A 64-year-old woman (patient #8) pathologically confirmed with retroperitoneal well-differentiated liposarcoma received radical operation 4 years ago. [^18^F]FDG PET/CT (**a**) demonstrated pelvic wall metastatic foci with intensive metabolic activity. But the abdominal wall foci and large pelvic metastatic foci showed no intensive uptake of [^18^F]FDG. Compared with [^18^F]FDG, [^68^ Ga]Ga-DOTA-FAPI-04 PET/CT (**b**) detected all the metastatic lesions with intense [^68^ Ga]Ga-DOTA-FAPI-04 activity. Black and white arrows indicated the tumor lesions detected by both tracers, and red arrows indicated the tumor lesions detected by [^68^ Ga]Ga-DOTA-FAPI-04 alone

**Fig. 4:**
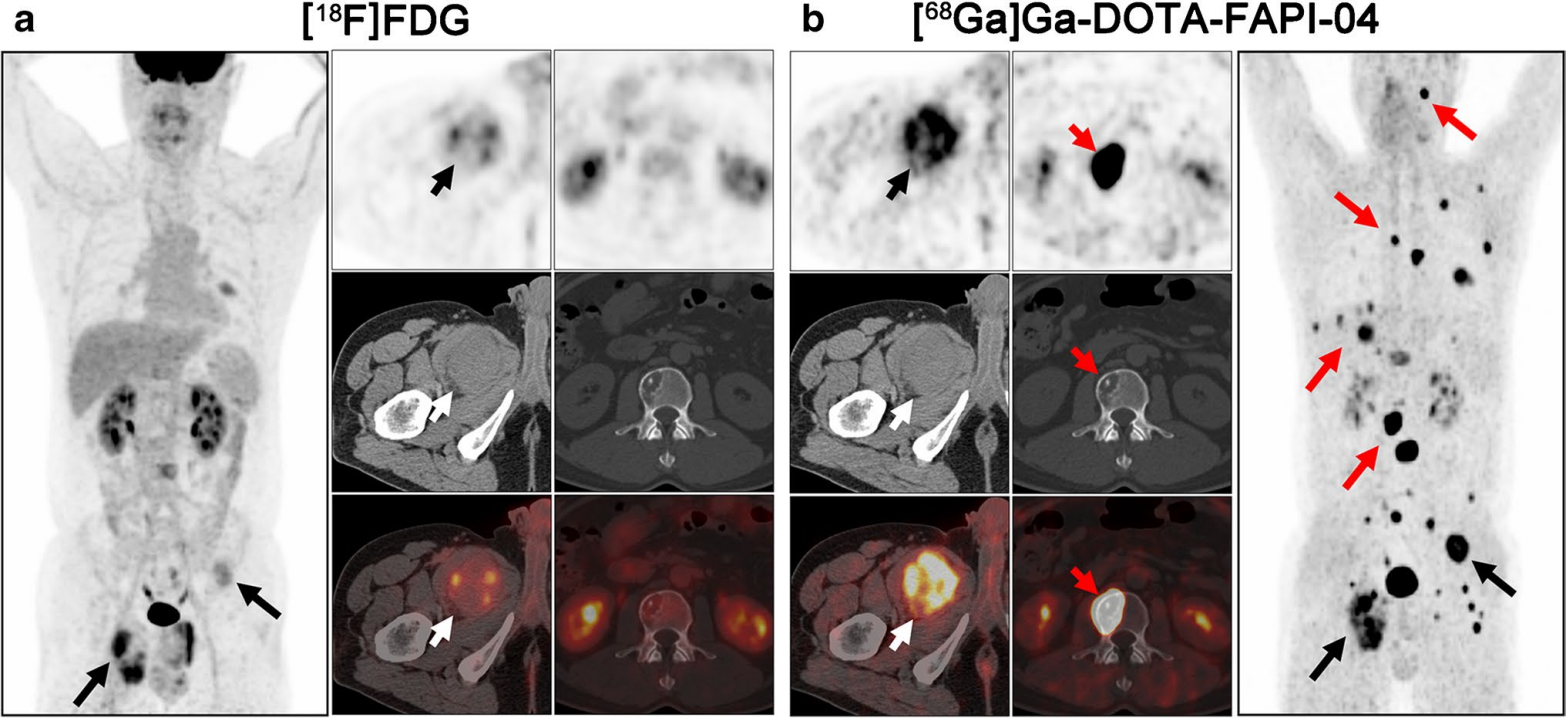
A 47-year-old man (patient #25) pathologically confirmed with malignant solitary fibrous tumor (MSFT) arising from right the thigh received radical operation 1 year ago. [^18^F]FDG PET/CT (**a**) demonstrated the relapse of right thigh and some bone metastases with low metabolic activity. Compared with [^18^F]FDG, [^68^ Ga]Ga-DOTA-FAPI-04 PET/CT (**b**) demonstrated more metastases, including lung, bone, and liver metastases. Moreover, all the relapse and metastases showed intensive uptake of [^68^ Ga]Ga-DOTA-FAPI-04. Black and white arrows indicated the tumor lesions detected by both tracers, and red arrows indicated the tumor lesions detected by [^68^ Ga]Ga-DOTA-FAPI-04 alone

**Fig. 5:**
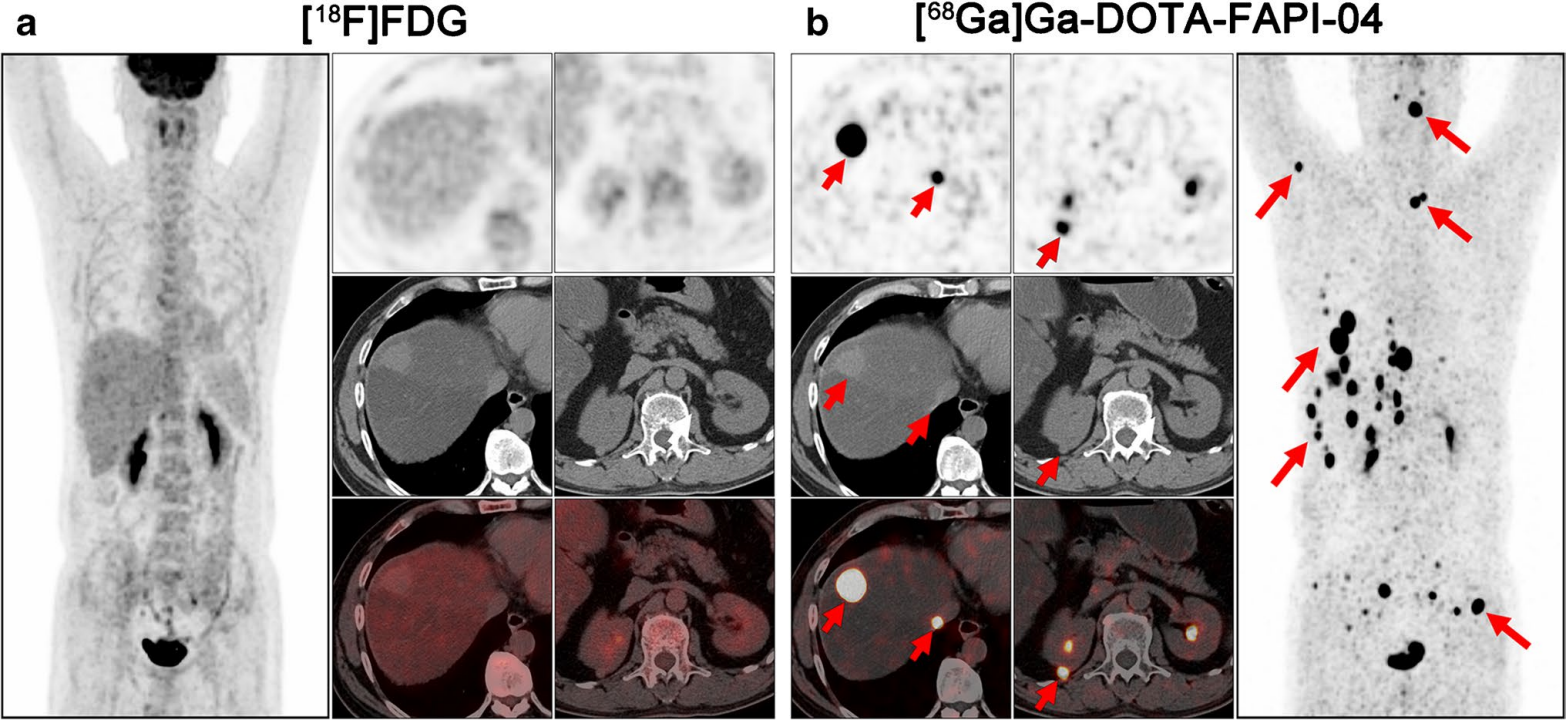
A 48-year-old man (patient #45) pathologically confirmed with liver interdigitating dendritic cell sarcoma (IDCS) received radical operation 2 years ago. [^18^F]FDG PET/CT (**a**) demonstrated none of the [^18^F]FDG-avid lesions. Interestingly, [^68^ Ga]Ga-DOTA-FAPI-04 PET/CT (**b**) demonstrated all the metastatic lesions with intense [^68^ Ga]Ga-DOTA-FAPI-04 activity, including liver, right kidney, and bone metastases. Red arrows indicated the tumor lesions detected by [^68^ Ga]Ga-DOTA-FAPI-04 alone

The representative figures of the 13 subtypes of recurrent STS are presented in Fig. 2. Overall, 282 local relapses and distant metastases were identified among the 45 patients. Among these lesions, 13 were verified by biopsy and 269 were assessed by follow-up imaging. Interestingly, [^68^ Ga]Ga-DOTA-FAPI-04 PET/CT led to upstaging in 4 out of 45 (8.89%) patients compared with [^18^F]FDG PET/CT (Table 1). In terms of different subtypes of recurrent STS, liposarcoma (Fig. 3), malignant solitary fibrous tumor (MSFT, Fig. 4), and interdigitating dendritic cell sarcoma (IDCS, Fig. 5) showed elevated uptake of [^68^ Ga]Ga-DOTA-FAPI-04, and demonstrated significantly higher semiquantitative values of SUV_max_ and TBR than [^18^F]FDG (*P* = 0.011, < 0.001, and < 0.001 for SUV_max_, respectively; *P* < 0.001, < 0.001, and < 0.001 for TBR, respectively; Table 2 and Table S1). Whereas, mean SUV_max_ and TBR presented favorable uptake of [^18^F]FDG over [^68^ Ga]Ga-DOTA-FAPI-04 in undifferentiated pleomorphic sarcoma (UPS) and rhabdomyosarcoma (RMS) (*P* = 0.003 and < 0.001 for SUV_max_, respectively; *P* < 0.001 and < 0.001 for TBR, respectively; Table 2 and Table S1). For the other eight subtypes of recurrent STS, [^68^ Ga]Ga-DOTA-FAPI-04 had similar performance in assessing local relapse and distant metastasis with [^18^F]FDG PET/CT.

**Table 2:** Comparison of SUV_max_ detected on [^18^F]FDG and [^68^ Ga]Ga-DOTA-FAPI-04 PET/CT in different subtypes of recurrent STS

Histology	No. of patients			[^18^F]FDG								[^68^ Ga]Ga-DOTA-FAPI-04			*P* value
		*Mean SUV_max_	SD		Range	95% *CI*	No. of lesions		*Mean SUV_max_	SD	Range		95% *CI*	No. of lesions	
UPS	7	10.81	4.94		2.20–27.49	9.49, 12.14	56		9.04	5.76	1.40–37.90		7.50, 10.58	56	0.003
Liposarcoma	6	8.08	8.22		1.60–22.65	6.67, 9.49	41		11.98	9.99	1.65–47.50		9.02, 14.95	46	0.011
Synovial sarcoma	6	6.68	3.74		1.60–15.20	4.17, 9.19	11		4.57	2.18	2.70–10.20		3.18, 6.00	12	0.339
RMS	5	6.89	2.09		2.80–12.40	6.08, 7.70	28		4.61	1.80	2.10–8.20		3.93, 5.30	29	< 0.001
MSFT	4	4.61	1.66		2.57–8.07	3.50, 5.72	11		15.06	13.32	2.07–78.48		11.55, 18.56	58	< 0.001
Ewing sarcoma	4	3.68	1.84		2.40–6.40	0.75, 6.60	4		4.78	3.44	2.60–9.90		− 0.69, 10.24	4	0.250
Leiomyosarcoma	4	4.03	0.90		2.90–5.10	3.28, 4.79	8		4.01	1.18	2.10–5.40		3.03, 5.00	8	0.922
Myxofibrosarcoma	3	3.64	0.45		3.21–4.10	2.53, 4.75	3		4.97	3.15	3.00–8.60		− 2.86, 12.79	3	0.999
ASPS	2	5.56	1.75		2.90–8.00	4.10, 7.03	8		6.11	2.45	2.30–9.40		4.07, 8.16	8	0.742
Epithelioid sarcoma	1	12.90	/		/	/	1		6.70	/	/		/	1	/
Aggressive fibromatosis	1	2.95	0.53		2.50–3.50	2.11, 3.79	4		7.30	0.62	6.50–8.00		6.32, 8.28	4	0.125
FDCS	1	5.70	3.20		1.90–10.70	3.55, 7.85	11		4.33	1.88	2.30–8.00		2.89, 5.78	9	0.123
IDCS	1	/	/		/	/	0		31.53	29.58	4.90–119.30		21.66, 41.39	37	< 0.001
Low grade (G1)	5	4.88	2.60		2.20–8.98	2.71, 7.06	8		13.49	8.56	2.50–28.60		9.09, 17.89	17	< 0.001
High grade (G2 + G3)	34	8.29	4.51		1.60–27.49	7.58, 9.00	158		9.45	9.51	1.40–78.48		8.12, 10.79	198	0.044

^*^SUV_max_ for only one lesion. *STS*, soft tissue sarcoma; *SD*, standard deviation; *CI*, confidence intervals; *UPS*, undifferentiated pleomorphic sarcoma; *RMS*, rhabdomyosarcoma; *MSFT*, malignant solitary fibrous tumor; *ASPS*, alveolar soft part sarcoma; *FDCS*, follicular dendritic cell sarcoma; *IDCS*, interdigitating dendritic cell sarcoma

Regarding the different grades, both low-grade and high-grade STS showed significantly higher uptake of [^68^ Ga]Ga-DOTA-FAPI-04 than [^18^F]FDG (*P* < 0.001 and = 0.044 for SUV_max_, respectively; *P* < 0.001 and = 0.023 for TBR, respectively; Table 2 and Table S1). Moreover, [^18^F]FDG uptake was higher for high-grade STS compared to low-grade STS. Conversely, low-grade STS showed higher uptake of [^68^ Ga]Ga-DOTA-FAPI-04 than high-grade STS.

### Comparison of [^18^F]FDG and [^68^ Ga]Ga-DOTA-FAPI-04 PET/CT based on different tissues and organs

**Fig. 6:**
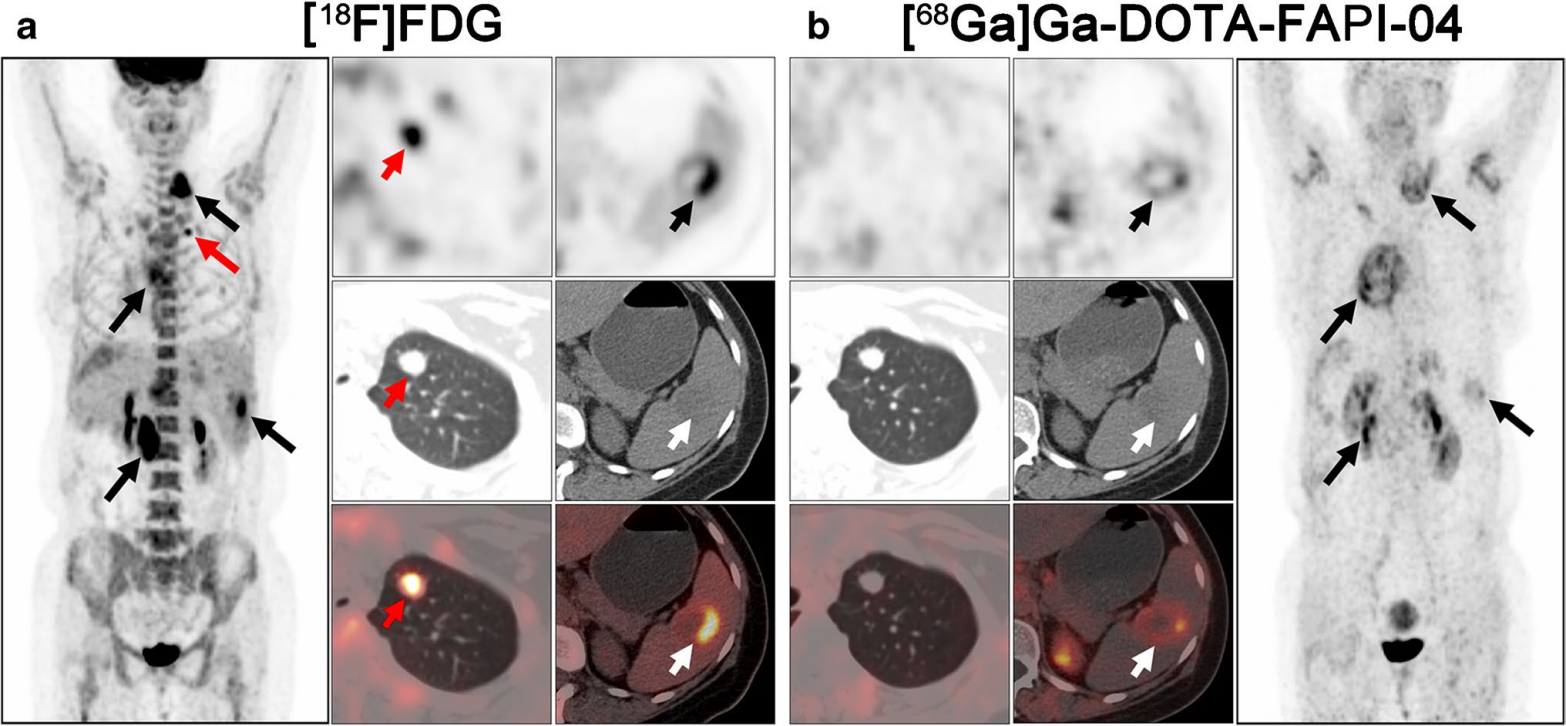
A 44-year-old woman (patient #44) pathologically confirmed with liver follicular dendritic cell sarcoma (FDCS) received radical operation 6 months ago. [^18^F]FDG PET/CT (**a**) demonstrated all the metastatic lesions with intense [^18^F]FDG activity, including liver, spleen, lung, and lymph node metastases. Compared with [^18^F]FDG, [^68^ Ga]Ga-DOTA-FAPI-04 PET/CT (**b**) demonstrated the liver, spleen, and lymph node metastases with moderate [^68^ Ga]Ga-DOTA-FAPI-04 activity. But no intensive [^68^ Ga]Ga-DOTA-FAPI-04 uptake was observed on the lung metastatic lesions. Black and white arrows indicated the tumor lesions detected by both tracers, and red arrows indicated the tumor lesions detected by [^18^F]FDG alone

The metastatic lesions were identified in the soft tissues (including muscle, pleura, and peritoneum), lung (Fig. 6), liver, bone, lymph node, spleen, pancreas, and kidney. Regarding different tissues and organs, soft tissues, liver, and bone metastases revealed intensive uptake of [^68^ Ga]Ga-DOTA-FAPI-04 and presented significantly higher semiquantitative values of SUV_max_ and TBR than [^18^F]FDG (*P* < 0.001, < 0.001, and < 0.001 for SUV_max_, respectively; *P* = 0.015, < 0.001, and < 0.001 for TBR, respectively; Table 3 and Table S2). Whereas, mean SUV_max_ and TBR presented favorable uptake of [^18^F]FDG over [^68^ Ga]Ga-DOTA-FAPI-04 in lymph node metastases (mean SUVmax = 8.57 ± 4.45 vs. 6.26 ± 4.06, *P* = 0.006; mean TBR = 11.71 ± 3.39 vs. 8.08 ± 3.95, *P* = 0.011; Table 3 and Table S2).

**Table 3:** Comparison of SUV_max_ detected on [^18^F]FDG and [^68^ Ga]Ga-DOTA-FAPI-04 PET/CT in different tissues and organs

Tissues and organs	Total lesions			[^18^F]FDG								[^68^ Ga]Ga-DOTA-FAPI-04			*P* value
		*Mean SUV_max_	SD		Range	95% *CI*	No. of lesions		*Mean SUV_max_	SD	Range		95% *CI*	No. of lesions	
Soft tissues	80	7.35	5.31		1.60–25.20	6.07, 8.62	69		10.74	9.38	2.10–47.50		8.66, 12.83	80	< 0.001
Lung	29	7.47	3.87		2.90–16.98	5.80, 9.14	23		9.09	8.03	1.40–34.07		5.53, 12.65	22	0.696
Liver	35	6.73	3.06		2.30–11.97	4.67, 8.79	11		21.60	27.73	2.70–119.30		12.07, 31.12	35	< 0.001
Bone	116	8.47	4.27		2.57–27.49	7.40, 9.54	63		13.03	15.61	2.40–90.30		10.16, 15.91	116	< 0.001
Lymph node	16	8.57	2.45		4.90–12.40	7.22, 9.93	15		6.26	4.06	2.30–19.65		4.10, 8.42	16	0.006
Spleen	3	4.30	2.82		1.90–7.40	− 2.70, 11.30	3		3.70	1.04	2.50–4.40		1.11, 6.29	3	0.999
Pancreas	2	7.97	/		7.50–8.43	/	2		5.80	/	3.30–8.30		/	2	/
Kidney	1	/	/		/	/	0		25.40	/	/		/	1	/
Sum	282	7.76	4.45		1.60–27.49	7.12, 8.41	186		12.64	15.67	1.40–119.30		10.78, 14.50	275	< 0.001

^*^SUV_max_ for only one lesion. *SD*, standard deviation; *CI*, confidence intervals

[^68^ Ga]Ga-DOTA-FAPI-04 outperformed [^18^F]FDG PET/CT in terms of the sensitivity, specificity, PPV, NPV, and accuracy for the diagnosis of recurrent lesions (*P* < 0.001, Table 4), and demonstrated significantly higher values of SUV_max_ and TBR (mean SUV_max_ = 12.64 ± 15.67 vs. 7.76 ± 4.45, *P* < 0.001; mean TBR = 19.03 ± 31.95 vs. 10.23 ± 6.62, *P* < 0.001).

**Table 4:** Lesion-based statistical analysis of diagnostic performance in [^18^F]FDG and [^68^ Ga]Ga-DOTA-FAPI-04 PET/CT

Tracer	Sensitivity (95% *CI*)	Specificity (95% *CI*)	PPV (95% *CI*)	NPV (95% *CI*)	Accuracy (95% *CI*)
[^18^F]FDG	65.96 (60.25–71.24)	21.43 (10.21–39.54)	89.42 (84.51–92.91)	5.88 (2.72–12.24)	61.94 (56.42–67.16)
[^68^ Ga]Ga-DOTA-FAPI-04	97.52 (94.97–98.79)	60.71 (42.41–76.43)	96.15 (93.25–97.84)	70.88 (50.83–85.09)	94.19 (91.01–96.30)

The data was presented as percentage (%). *STS*, soft tissue sarcoma; *CI*, confidence intervals; *PPV*, positive predictive value; *NPV*, negative predictive value

## Discussion

This prospective study of 45 patients with 13 subtypes of recurrent STS suggests that [^68^ Ga]Ga-DOTA-FAPI-04 PET/CT is a promising new imaging modality for recurrent surveillance of STS and provides an enhancement to [^18^F]FDG PET/CT.

STS represents a distinct group of rare malignant tumors with high heterogeneity, which remains a major concern in cancer management [18–20]. Several previous studies [4, 21, 22] reported the usefulness of [^18^F]FDG PET/CT in the diagnosis of primary sarcomas, particularly in high-grade sarcomas. Nevertheless, various subtypes of STS exhibiting a spectrum of atypical imaging appearances may lead to challenge in recurrent surveillance of STS by [^18^F]FDG PET/CT [8]. In a prospective trial of 41 patients with clinically suspected tumor relapse of STS, [^18^F]FDG PET/MRI demonstrated higher sensitivity and accuracy and lower specificity for the detection of local tumor recurrence than MRI alone (95.0% vs. 80.0%, 89.5% vs. 80.7%, and 76.5% vs. 82.4%, respectively) [23]. However, in the present study, [^18^F]FDG PET/CT detected approximately two-thirds of recurrent lesions with a sensitivity of 65.96%, a specificity of 21.43%, and an accuracy of 61.94% in the 13 subtypes of STS. Compared to [^18^F]FDG, [^68^ Ga]Ga-DOTA-FAPI-04 PET/CT identified almost all lesions (275/282) and presented significant improved sensitivity, specificity, and accuracy (97.52%, 60.71%, and 95.15%, respectively; *P* < 0.001). Furthermore, high PPV for [^68^ Ga]Ga-DOTA-FAPI-04 PET/CT was also observed in the present study, which is consistent with the results from Kessler et al. (96.15% vs. 97%) [24]. Moreover, the higher sensitivity and accuracy of [^68^ Ga]Ga-DOTA-FAPI-04 over [^18^F]FDG PET/CT could bring benefit in accurately restaging (upstaging in 4 out of 45 patients in the present study and 8 out of 43 patients in Kessler et al. study) and guiding the treatment decision in recurrent STS [24]. Prominent higher uptake of [^68^ Ga]Ga-DOTA-FAPI-04 than [^18^F]FDG was observed in recurrent lesions of STS in terms of mean SUV_max_ and TBR (*P* < 0.001), which is in line with previous studies [9, 25]. It should be noted that the intensive uptake of [^68^ Ga]Ga-DOTA-FAPI-04 presenting in wound healing, uterus, arthritis, and periodontitis may be misdiagnosed as local relapse or distant metastasis. This is caused by fibrotic activity in these conditions [26]. Thus, more researches focused on non-tumor–specific uptake of FAPI are still needed [27, 28].

In a recent study, Koerber el al. [25] reported the imaging of seven subtypes of bone and soft tissue sarcoma by FAPI-PET/CT in fifteen patients, demonstrating the high uptake of FAPI for high-grade sarcomas and low uptake for low-grade sarcomas. However, the patient cohort and tumor subtype are small, and the compounds of FAPI are variance (FAPI-04, FAPI-46, and FAPI-74). These factors may result in data bias. In line with previous studies [4, 21], higher [^18^F]FDG uptake was also observed in high-grade STS compared to low-grade STS in the present study. However, [^68^ Ga]Ga-DOTA-FAPI-04 uptake was lower in high-grade STS than low-grade STS (mean SUV_max_ = 9.45 vs. 13.49) in the present study, which is not consistent with Koerber’s research [25]. This may be caused by that the most cases included in the low-grade group were well-differentiated liposarcoma (3/5) and MSFT (1/5), which showed prominent expression of FAP on tumor cell surface [12]. Nevertheless, a significantly higher uptake of [^68^ Ga]Ga-DOTA-FAPI-04 was found for high-grade STS compared to [^18^F]FDG (mean SUV_max_ = 9.45 vs. 8.29, *P* = 0.044), indicating the potential role of [^68^ Ga]Ga-DOTA-FAPI-04 PET/CT in detecting recurrent lesions with distinct visual discrimination regardless of tumor grade.

With respect to the diagnostic performance of different relapsed and metastatic lesions, [^68^ Ga]Ga-DOTA-FAPI-04 PET/CT outperformed [^18^F]FDG PET/CT in soft tissues, liver, bone, and kidney metastases with improved tumor retention and low background uptake. This advantage of [^68^ Ga]Ga-DOTA-FAPI-04 PET/CT over [^18^F]FDG PET/CT was also demonstrated in many other types of cancer, including head and neck cancers [29], hepatic carcinoma [30], and gastrointestinal cancers [31]. The absence of [^68^ Ga]Ga-DOTA-FAPI-04 in normal organs and tissues (e.g., liver, bone, and intestines) will benefit imaging of liver, bone, and abdomen metastases with higher tumor-to-background contrast and better lesion delineation than [^18^F]FDG PET/CT. However, it should be noted that [^68^ Ga]Ga-DOTA-FAPI-04 was false negative in 7 out of 29 lung metastases (4 from patient #34 and 3 from patient #44, Fig. 6). In a recent animal-based study, Ding et al. [32] found that the expression of FAP was prominent in lung metastatic lesion at the early stage but descended during the progress of tumor metastasis. Thus, the diagnostic performance of [^68^ Ga]Ga-DOTA-FAPI-04 PET in detecting lung metastasis remains uncertain in recurrent STS.

Despite advances in chemotherapy, targeted therapy and immunotherapy over the last decades, the prognosis for patients with metastatic STS remains poor [33]. Limited options with clinical efficacy for the metastatic or local advanced STS existed in addition to standard treatment [2]. Recently, Kratochwil et al. [34] reported a case of metastatic sarcoma treated with ^90^Y/^153^Sm-labeled FAPI-46 achieving stable disease for 8 months. Moreover, Ferdinandus et al. [35] demonstrated the potential role of FAP–targeted radioligand therapy in a study of nine patients with solid tumors. Surprisingly, disease control was observed in three patients with sarcomas and one patient with pancreatic ductal adenocarcinoma. These studies indicated that FAP–targeted radioligand therapy may present as a novel promising treatment strategy for incurable recurrent STS. Thus, non-invasive selection of the suitable patients with STS for the coming FAP–targeted radioligand therapy will emerge as a critical issue, and our work serves as a foundation for that.

The major limitation of this study is the relatively low number of patients and limited subtypes of STS. As STS is a large group of malignant tumors, it is hard to enroll all subtypes in a single center. Thus, larger multi-center studies containing more subtypes of STS are still needed to be carried out in the future. Another limitation is that not all [^68^ Ga]Ga-DOTA-FAPI-04 and [^18^F]FDG positive lesions are pathologically confirmed and examined FAP expression. Nevertheless, these lesions are also verified by continuous follow-up. Furthermore, a positive correlation with [^68^ Ga]Ga-DOTA-FAPI-04 uptake and FAP expression is reported in previous study [30].

## Conclusion

The current study demonstrated that [^68^ Ga]Ga-DOTA-FAPI-04 PET/CT is a promising new imaging modality for recurrent surveillance of STS regardless of tumor grade. [^68^ Ga]Ga-DOTA-FAPI-04 compared favorably with [^18^F]FDG PET/CT for identifying recurrent lesions of liposarcoma, MSFT, and IDCS. For UPS and RMS, [^18^F]FDG showed a superior diagnostic efficacy than [^68^ Ga]Ga-DOTA-FAPI-04 PET/CT. Moreover, [^68^ Ga]Ga-DOTA-FAPI-04 had similar performance in assessing recurrent surveillance of synovial sarcoma, Ewing sarcoma, leiomyosarcoma, myxofibrosarcoma, alveolar soft part sarcoma (ASPS), aggressive fibromatosis, and follicular dendritic cell sarcoma (FDCS) with [^18^F]FDG PET/CT. The clinical value of FAP–targeted radioligand therapy in recurrent STS should be further investigated.

## Supplementary Information

Below is the link to the electronic supplementary material.

Supplementary file1 (DOCX 30 KB)

## Data Availability

The datasets used and analyzed during the current study are available from the corresponding author on reasonable request.
