# Supplementary material for: Head-to-head evaluation of [18F]FDG and [68 Ga]Ga-DOTA-FAPI-04 PET/CT in recurrent soft tissue sarcoma
Source: Eur J Nucl Med Mol Imaging. 2022 Feb 3;49(8):2889–901. doi: 10.1007/s00259-022-05700-4 (PMC9206606; doi:10.1007/s00259-022-05700-4)
Supplement: Supplementary file 1 — Supplementary file1 (DOCX 30 KB) [file 259_2022_5700_MOESM1_ESM.docx]

**Head-to-head Evaluation of [^18^F]FDG and [^68^Ga]Ga-DOTA-FAPI-04 PET/CT in** **Recurrent Soft Tissue Sarcoma**

Bingxin Gu^1^ • Xin Liu^2^ • Shuoer Wang^3^ • Xiaoping Xu^1^ • Xiaosheng Liu • Silong Hu • Wangjun Yan^3^ • Zhiguo Luo^2^ • Shaoli Song^1^

^1^ Department of Nuclear Medicine, Fudan University Shanghai Cancer Center; Department of Oncology, Shanghai Medical College, Fudan University; Center for Biomedical Imaging, Fudan University; Shanghai Engineering Research Center of Molecular Imaging Probes; Key Laboratory of Nuclear Physics and Ion-beam Application (MOE), Fudan University; Shanghai, China

^2^ Department of Medical Oncology, Fudan University Shanghai Cancer Center, Shanghai, China

^3^ Department of Musculoskeletal Tumor, Fudan University Shanghai Cancer Center, Shanghai, China

**Bingxin Gu and Xin Liu contributed equally to this work.**

**Correspondence to**: Zhiguo Luo, MD, luozhiguo88@163.com, and Shaoli Song, MD, [shaoli-song@163.com](mailto:shaoli-song@163.com)

**Funding information** This work was funded by the National Key Research and Development Program of China (Grant number 2020YFA0909000), National Natural Science Foundation of China (Grant number 81771861, 81971648, and 81901778) and Shanghai Anticancer Association Program (Grant number HYXH2021004).

**European Journal of Nuclear Medicine and Molecular Imaging**

**Table S1.** Comparison of TBR detected on [^18^F]FDG and [^68^Ga]Ga-DOTA-FAPI-04 PET/CT in different subtypes of recurrent STS

| Histology | No. of patients |  | [^18^F]FDG | | | |  |  | [^68^Ga]Ga-DOTA-FAPI-04 | | | | *P* value |
| --- | --- | --- | --- | --- | --- | --- | --- | --- | --- | --- | --- | --- | --- |
|  |  | *Mean TBR | SD | Range | 95% CI | No. of lesions |  | *Mean TBR | SD | Range | 95% CI | No. of lesions |  |
| UPS | 7 | 15.30 | 7.19 | 1.03-39.27 | 13.37, 17.22 | 56 |  | 9.75 | 6.37 | 3.19-42.11 | 8.05, 11.46 | 56 | <0.001 |
| Liposarcoma | 6 | 8.22 | 5.89 | 2.27-32.36 | 6.36, 10.08 | 41 |  | 17.15 | 14.43 | 2.50-68.14 | 12.86, 21.43 | 46 | <0.001 |
| Synovial sarcoma | 6 | 6.82 | 5.42 | 1.33-19.00 | 3.18, 10.46 | 11 |  | 5.18 | 2.76 | 3.31-12.75 | 3.43, 6.93 | 12 | 0.850 |
| RMS | 5 | 9.40 | 3.30 | 3.50-17.71 | 8.12, 10.67 | 28 |  | 7.37 | 3.13 | 2.56-13.67 | 6.18, 8.56 | 29 | 0.001 |
| MSFT | 4 | 8.01 | 4.95 | 3.67-21.00 | 4.69, 11.33 | 11 |  | 16.38 | 14.82 | 2.08-87.20 | 12.48, 20.27 | 58 | <0.001 |
| Ewing sarcoma | 4 | 6.63 | 3.25 | 3.43-10.67 | 1.46, 11.80 | 4 |  | 8.09 | 5.88 | 3.71-16.50 | -1.28, 17.44 | 4 | 0.750 |
| Leiomyosarcoma | 4 | 6.08 | 2.15 | 2.32-9.80 | 4.28, 7.88 | 8 |  | 6.21 | 3.32 | 3.39-13.50 | 3.43, 8.98 | 8 | 0.770 |
| Myxofibrosarcoma | 3 | 4.98 | 1.93 | 3.73-7.20 | 0.19, 9.77 | 3 |  | 5.52 | 3.50 | 3.33-9.56 | -3.18, 14.21 | 3 | 0.999 |
| ASPS | 2 | 8.63 | 2.87 | 4.14-13.33 | 6.23, 11.02 | 8 |  | 11.46 | 5.22 | 3.83-18.80 | 7.09, 15.82 | 8 | 0.195 |
| Epithelioid sarcoma | 1 | 21.50 | / | / | / | 1 |  | 11.17 | / | / | / | 1 | / |
| Aggressive fibromatosis | 1 | 4.92 | 0.88 | 4.17-5.83 | 3.52, 6.31 | 4 |  | 9.13 | 0.77 | 8.13-10.00 | 7.90, 10.35 | 4 | 0.125 |
| FDCS | 1 | 7.53 | 7.10 | 1.06-25.00 | 2.76, 12.30 | 11 |  | 6.00 | 2.22 | 2.56-8.89 | 4.30, 7.70 | 9 | 0.520 |
| IDCS | 1 | / | / | / | / | 0 |  | 64.38 | 67.12 | 7.00-298.30 | 42.00, 86.76 | 37 | <0.001 |
| Low grade (G1) | 5 | 5.45 | 2.99 | 2.27-9.98 | 3.00, 7.95 | 8 |  | 16.53 | 12.78 | 2.08-40.13 | 10.00, 23.10 | 17 | <0.001 |
| High grade (G1 and G2) | 34 | 10.93 | 6.68 | 1.03-39.27 | 9.88, 11.98 | 158 |  | 11.88 | 11.38 | 2.50-87.20 | 10.28, 13.47 | 198 | 0.023 |

*TBR for only one lesion. Abbreviations: TBR = tumor-to-background ratio; STS = soft tissue sarcoma; SD = standard deviation; CI = confidence intervals; UPS = undifferentiated pleomorphic sarcoma; RMS = rhabdomyosarcoma; MSFT = malignant solitary fibrous tumor; ASPS = alveolar soft part sarcoma; FDCS = follicular dendritic cell sarcoma; IDCS = interdigitating dendritic cell sarcoma.

**Table S2.** Comparison of TBR detected on [^18^F]FDG and [^68^Ga]Ga-DOTA-FAPI-04 PET/CT in different tissues and organs

| Tissues and organs | Total lesions |  | | | | [^18^F]FDG | |  |  | | | [^68^Ga]Ga-DOTA-FAPI-04 | | | *P* value |
| --- | --- | --- | --- | --- | --- | --- | --- | --- | --- | --- | --- | --- | --- | --- | --- |
|  |  | *Mean TBR | SD | Range | 95% CI | | No. of lesions |  | *Mean TBR | SD | Range | | 95% CI | No. of lesions |  |
| Soft tissues | 80 | 9.97 | 7.51 | 1.03-36.00 | 8.17, 11.78 | | 69 |  | 12.42 | 10.54 | 2.56-45.88 | | 10.08, 14.77 | 80 | 0.015 |
| Lung | 29 | 10.55 | 5.51 | 3.58-25.00 | 8.17, 12.93 | | 23 |  | 18.94 | 16.06 | 3.30-68.14 | | 11.82, 26.06 | 22 | 0.063 |
| Liver | 35 | 2.65 | 0.81 | 1.35-4.18 | 2.11, 3.19 | | 11 |  | 50.79 | 71.18 | 3.30-298.30 | | 26.34, 75.24 | 35 | <0.001 |
| Bone | 116 | 11.71 | 6.28 | 3.23-39.27 | 10.13, 13.29 | | 63 |  | 15.93 | 20.86 | 2.08-129.00 | | 12.09, 19.77 | 116 | <0.001 |
| Lymph node | 16 | 11.71 | 3.39 | 6.89-17.71 | 9.84, 13.59 | | 15 |  | 8.08 | 3.95 | 2.56-17.86 | | 5.97, 10.18 | 16 | 0.011 |
| Spleen | 3 | 2.39 | 1.56 | 1.06-4.11 | -1.50, 6.28 | | 3 |  | 6.17 | 1.74 | 4.17-7.33 | | 1.84, 10.49 | 3 | 0.250 |
| Pancreas | 2 | 11.38 | / | 10.71-12.04 | / | | 2 |  | 7.36 | / | 5.50-9.22 | | / | 2 | / |
| Kidney | 1 | / | / | / | / | | 0 |  | 36.29 | / | / | | / | 1 | / |
| Sum | 282 | 10.23 | 6.62 | 1.03-39.27 | 9.28, 11.19 | | 186 |  | 19.03 | 31.95 | 2.08-298.30 | | 15.24, 22.83 | 275 | <0.001 |

*TBR for only one lesion. Abbreviations: TBR = tumor-to-background ratio; SD = standard deviation; CI = confidence intervals.
